# Multiplex analysis of 40 cytokines do not allow separation between endometriosis patients and controls

**DOI:** 10.1038/s41598-019-52899-8

**Published:** 2019-11-13

**Authors:** Tamara Knific, Dmytro Fishman, Andrej Vogler, Manuela Gstöttner, René Wenzl, Hedi Peterson, Tea Lanišnik Rižner

**Affiliations:** 1 Institute of Biochemistry, Faculty of Medicine, University of Ljubljana, 1000 Ljubljana, Slovenia; 2 Institute of Computer Science, University of Tartu, Liivi 2, 50409 Tartu, Estonia; 3 Quretec Ltd., Ülikooli 6A, Tartu, 51003 Estonia; 4 Department of Obstetrics and Gynaecology, University Medical Centre Ljubljana, 1000 Ljubljana, Slovenia; 5 Department of Obstetrics and Gynecology, Medical University Vienna, 1090 Vienna, Austria

**Keywords:** Diagnostic markers, Diagnostic markers

## Abstract

Endometriosis is a common gynaecological condition characterized by severe pelvic pain and/or infertility. The combination of nonspecific symptoms and invasive laparoscopic diagnostics have prompted researchers to evaluate potential biomarkers that would enable a non-invasive diagnosis of endometriosis. Endometriosis is an inflammatory disease thus different cytokines represent potential diagnostic biomarkers. As panels of biomarkers are expected to enable better separation between patients and controls we evaluated 40 different cytokines in plasma samples of 210 patients (116 patients with endometriosis; 94 controls) from two medical centres (Slovenian, Austrian). Results of the univariate statistical analysis showed no differences in concentrations of the measured cytokines between patients and controls, confirmed by principal component analysis showing no clear separation amongst these two groups. In order to validate the hypothesis of a more profound (non-linear) differentiating dependency between features, machine learning methods were used. We trained four common machine learning algorithms (decision tree, linear model, k-nearest neighbour, random forest) on data from plasma levels of proteins and patients’ clinical data. The constructed models, however, did not separate patients with endometriosis from the controls with sufficient sensitivity and specificity. This study thus indicates that plasma levels of the selected cytokines have limited potential for diagnosis of endometriosis.

## Introduction

Endometriosis is a common benign gynaecological disease where endometrium like tissue is displaced and found outside the uterine cavity at ectopic locations. Endometriosis affects mainly women of reproductive age and is associated with pelvic pain and infertility^1,2^. Based on location of endometriotic lesions three types of endometriosis can be defined: ovarian, peritoneal and deep infiltrating endometriosis^3,4^. Laparoscopic visualization of the lesions followed by histological examination enables confirmation of endometriosis and according to the extent and location of lesions the disease is classified into four stages (minimal, mild, moderate and severe). The combination of non-specific symptoms and invasive laparoscopic procedure needed for the definitive diagnosis, results in up to 10 years of delay from the start of the symptoms to the definitive diagnosis of endometriosis^1,5^. Although several theories have been proposed that attempt to explain reasons for the clinical manifestation of endometriosis (metaplasia, transplantation), Sampson’s theory of retrograde menstruation still remains most widely accepted^1,6–9^. This theory states that the blood containing endometrial cells flows through the fallopian tubes into the pelvic cavity during menstruation, leading to ectopic endometrial lesions. Endometriosis is also an oestrogen-dependent and chronic inflammatory disease. Thus, several additional factors, such as impaired immune system, genetic and epigenetic predispositions as well as environmental factors were shown to play a role in determining whether an individual will develop the condition^10^. Endometrial tissue, which is displaced at different parts of the peritoneal cavity, induces inflammation. Inflammation is a complex process which is regulated by cytokines, a vast and diverse group of proteins that have a key role in the proliferation, activation of B cells, adhesion and cell chemotaxis. Cytokines via inflammation can therefore influence the onset and progression of endometriosis^11^. These proteins include growth factors, interferons, interleukins (IL) and chemokines^12,13^. Chemokines are a small (8–10 kDa) group of pro-inflammatory polypeptides and signal proteins as they induce chemotaxis and are involved in the inflammatory response. Based on the distance between the first two cysteine residues chemokines can be divided into four groups; namely C (γ chemokines), CC (β chemokines), CXC (α chemokines), and CX3C (δ chemokines). The CXC group of chemokines can be further subdivided according to the presence/absence of ELR (glutamic acid-leucine-arginine) motif^14^. Since cytokines and chemokines can be released into the bloodstream their plasma/serum concentrations can easily be determined and thus represent potential biomarkers for the non-invasive diagnosis of endometriosis. There have been several thorough review papers published by May *et al*., Rižner, Gupta *et al*. and Nisenblat *et al*. describing potential biomarkers for endometriosis, reported from 1984 to 2015^15–18^. In addition, Borrelli *et al*. systematically reviewed published studies on chemokines as potential biomarkers of endometriosis where in total 27 different chemokines have been evaluated where the majority of the studies focused on the diagnostic potential of CXCL8, CCL2 and CCL5^19^. The authors of these systematic reviews emphasized the importance of employing high quality standardized procedures when evaluating biomarkers for the diagnosis of endometriosis. Starting from sample collection and storage to collecting more detailed clinical data. These reviews emphasized also a need for multicentre validation studies performed on an independent set of patients from different populations.

In our previous study we evaluated the concentrations of 16 cytokines and other secretory proteins in peritoneal fluid and serum samples from patients with ovarian endometriosis, benign ovarian cysts and healthy women^20^. In peritoneal fluid the models with the highest diagnostic accuracies included: (i) IL-8 and the ratio of ficolin2 to glycodelin (ii) the ratio of biglycan to leptin and also the ratio of RANTES to IL-6; both in combination with age; the model with the highest diagnostic accuracy had an area under the curve (AUC) of 0.9. In serum the best characteristics were shown for models including: (i) the ratio between leptin and glycodelin and (ii) the ratio between ficolin2 and glycodelin; again both in combination with age; where the models with the highest diagnostic accuracies had a slightly lower AUC of 0.86 and 0.85, respectively^20^. The present study was performed on a different set of patient samples that were collected from two medical centres (Slovenian, Austrian) and included evaluation of 40 different cytokines - mainly chemokines in plasma samples. We decided to evaluate a different set of proteins from aforementioned studies in order to broaden the set of potential biomarkers for further validation studies that could include previous, as well as potential novel biomarkers. To the best of our knowledge this is the first study that evaluated such a broad spectrum of inflammatory proteins in plasma samples from a large, well-defined group of patients with different types of endometriosis. Aims of the present study were therefore to evaluate whether a single cytokine or combination of cytokines in a large, well-defined patient population can differentiate patients with endometriosis from control patients. If we identified cytokines with diagnostic potential we planned to design a diagnostic model with sufficient sensitivity and specificity, based on the plasma concentrations of cytokines and gathered patients’ clinical data, and with the use of appropriate statistical and bioinformatics analysis.

## Materials and Methods

### Study design and sample source

The prospective case-control study was approved by both (i.e. Slovenian and Austrian) National Medical Ethics Committees (0120-127/2016-2 and EMMA 545/2010, respectively) and all the participants signed their written informed consent before being included in the study. Inclusion criteria comprised endometriosis-like symptoms (i.e. infertility and/or pain) as well as benign gynaecological conditions (i.e. different types of cysts and/or myomas). Exclusion criteria included pregnancy, age below 18 or above 50 years, menopausal status, gynaecological malignancies, other types of cancer, cancelled operation, HIV infection and the presence of haemolysis in plasma samples. The aim was to collect approximately 200 samples, with approximately one-to-one ratio of patients and controls to achieve more than 80% statistical power (probability to reject null hypothesis if it is false) and less than 5% Type I error rate under assumption that mean concentrations of cytokines noticeably differ between conditions.

**Figure 1:**
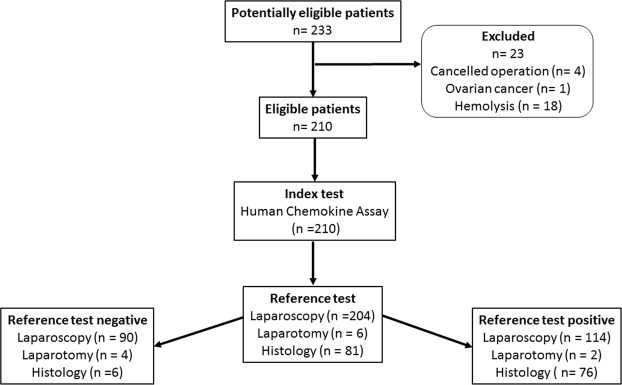
Flowchart of patient recruitment.

Patient enrolment took place from March 2013 to September 2016 at the Departments of Obstetrics and Gynaecology, University Medical Centre Ljubljana, Slovenia and the Medical University Vienna, Austria. At both Departments of Gynecology patients were recruited by senior gynecologists with the help of study nurses. Blood samples were analyzed in 2016. The time interval between recruitment/surgery and blood analysis (index test) was few weeks to 3 years. On the day of the surgery (Vienna) or one day to one week before surgery (Ljubljana) blood samples were collected according to a strict standard operating procedure. Blood samples of 4 ml were taken into BD Vacutainer tubes, (#368861, Becton Dickinson and Company, NJ, USA). Within one hour after collection the samples were centrifuged at 1400 g for 10 min at 4 °C. The plasma was aspirated and samples were aliquoted into 100 μL volumes and stored at −80 °C until analysis. Participants were interviewed regarding their ethnic origin, life style (i.e. diet, smoking status, sport and recreation, stress level), medical history especially with regards to different types of pain that are associated with endometriosis (pelvic pain, dysmenorrhea, dyschezia and dyspareunia) as well as medication intake a week prior to surgery, the use of oral contraceptives and hormonal therapy, current or in the three months prior to surgery. The intensity of dysmenorrhea and dyspareunia were evaluated using a validated visual analogue scale of 10 points. The reference test was laparoscopy (in exceptional cases laparotomy) with visualization of typical lesions and histological evaluation. Laparoscopy and laparotomy were performed by expert surgeons with at least ten years of experience. In total out of 233 patients 210 met inclusion criteria of whom 116 were laparoscopically (or by laparotomy) and histologically characterized by the presence of endometriosis and 94 by the absence of it (Table 1, Fig. 1).

**Table 1:** Clinical characteristics of the study participants (^§^Mann-Whitney test for continuous variables and Fisher’s or Chi-square test for categorical variables; P values calculated by comparing controls to patients with endometriosis); ns, not significant.

Characteristic	Subgroup	Controls n = 94		Patients with endometriosis n = 116		P-value^§^
		Frequency	[%]	Frequency	[%]	
Age (years)	<26	17	18.1	17	14.7	ns
	26–29.9	19	20.2	22	19.0	
	30–35.9	26	27.7	49	42.2	
	36–40.9	21	22.3	22	19.0	
	>41	11	11.7	6	5.2	
BMI (kg/m^2^)	<18.5	3	3.2	11	9.5	<0.05
	18.6–24.9	59	62.8	78	67.2	
	25–29.9	25	26.6	16	13.8	
	>30	7	7.4	11	9.5	
Smoking status	Nonsmoker	45	47.9	68	58.6	ns
	Smoker	31	33.0	29	25.0	
	Occasional smoker	5	5.3	6	5.2	
	Former smoker	12	12.8	12	10.3	
	Missing data	1	1.1	1	0.9	
Menstrual phase	Proliferative	41	43.6	49	42.2	ns
	Secretory	41	43.6	59	50.9	
	Anovulatory	2	2.1	0	0	
	Oral contraceptives	4	4.3	6	5.2	
	Missing data	6	6.4	2	1.7	
Hormonal therapy three months prior to surgery	No	86	91.5	104	89.7	ns
	Yes	8	8.5	12	10.3	
	Missing data	0	0	0	0	
Oral contraceptives three months prior to surgery	No	83	88.3	101	87.1	ns
	Yes	11	11.7	15	12.9	
	Missing data	0	0	0	0	
Medication intake a week prior to surgery	No	55	58.5	62	53.4	ns
	Yes	39	41.5	54	46.6	
	Missing data	0	0	0	0	
Additional pathologies/conditions Cysts	No	63	67.0	102	87.9	
						<0.01
	Yes	31	33.0	14	12.1	
Fallopian tube related	No	79	84.0	113	97.4	<0.01
	Yes	15	16.0	3	2.6	
Uterus related	No	86	91.5	107	92.2	ns
	Yes	8	8.5	9	7.8	
Adenomyosis	No	92	97.9	113	97.4	ns
	Yes	2	2.1	3	2.6	
Adhesions	No	80	85.1	89	76.7	ns
	Yes	14	14.9	27	23.3	
Inflammation related conditions	No	83	88.3	113	97.4	<0.05
	Yes	11	11.7	3	2.6	
Borderline ovarian tumour	No	92	97.9	116	100	ns
	Yes	2	2.1	0	0

Additional pathologies/conditions were identified after the surgical procedure. The phase of the menstrual cycle was estimated based on the date of the last menstruation and the thickness, as well as appearance, of the endometrium determined by ultrasound. The study was designed to meet the principles of the Declaration of Helsinki (Ethical Principles for Medical Research Involving Human Subjects), Oviedo Convention (Protecting Human Rights in the Biomedical Field) and the Code of Medical Ethics.

### Biomarker measurements

All methods were performed in accordance with the relevant guidelines and regulations. The Luminex xMAP multiplexing and the Bio-Plex Pro™ Human Chemokine Assay platforms (#171ak99mr2, lots: #64025638, #64040537 Bio-Rad Laboratories, CA, USA) were used according to the manufacturer’s protocol. Briefly, the method is based on 5.5 μm polystyrene beads that are labelled with two different fluorescent dyes in different ratios assigned for each individual antibody, thus enabling quantification of 40 different cytokines, mainly chemokines in each sample (Table 2). The intra assay and inter assay variability of the Human Chemokine Assay, as specified by the producer, was 2–6% CV and 2–8% CV, respectively. The samples were anonymized and the person performing the assays was blind to identity of the samples and the result of the surgery. According to the producers’ instruction manual plasma was diluted fourfold prior to analysis. Bio-Plex™ Manager Software with a 5-parameter logistic regression modelling was used to calculate final concentrations. Calibrations and verifications were performed prior to every analysis with the use of commercially available and recommended kits (MPX-PVER-K25, MPX-CAL-K25; Luminex, Austin, Texas, USA). Clinical data that were obtained from the patients (i.e. metadata) was included in the statistical modelling. The data were processed using Microsoft Excel 2003, and for statistical analysis we used GraphPad Prism Software version 5.00 for Windows (San Diego, CA, USA), R programming language^21^ version 3.4.3 (2017-11-30) – “Kite-Eating-Tree” and R Studio version 1.1.383 with packages such as *mice*, *caret* and *ggplot2*. Corrected P value of <0.05 was considered significant.

**Table 2:** Plasma concentrations of the measured cytokines in pg/mL (with no significant differences between patients with endometriosis and controls).

	Controls; n = 94			Patients with endometriosis; n = 116			Controls; n = 94				Patients with endometriosis; n = 116		
	Median	Mean ± SD	Range	Median	Mean ± SD	Range	Median		Mean ± SD	Range	Median	Mean ± SD	Range
CCL21	4812.6	5198.8 ± 1769.4	2491.1–11051.8	4966.4	5695.7 ± 2031.5	1870.2–14517.3	IL-16	339.3	347.6 ± 111.7	97.9–609.3	341.7	351.4 ± 128.7	82.4–967.4
CXCL13	17.4	26.1 ± 30.8	8.1–205.4	17.9	21.6 ± 18.4	3.6–157.5	CXCL10	26.6	32.7 ± 24.7	13.3–226.2	26.5	31.3 ± 20.7	7.8–170.7
CCL27	584.0	607.3 ± 239.3	82.8–1255.1	637.9	658.9 ± 265.2	172.7–1675.3	CXCL11	109.7	138.0 ± 122.5	12.1–1068.74	122.9	141.1 ± 79.8	44.2–657.8
CXCL5	165.1	168.4 ± 112.8	11.1–494.2	141.7	159.2 ± 108.8	6.83–440.6	CCL22	586.5	626.9 ± 258.6	203.3–2024.0	578.6	598.9 ± 205.6	149.5–1236.6
CCL11	76.0	78.2 ± 13.8	50.1–116.1	73.4	74.6 ± 13.0	46.5–104.6	MIF	955.4	1398.0 ± 1550.4	167.9–10307.6	830.0	1338.4 ± 1425.3	192.9–8217.7
CCL24	98.4	122.2 ± 99.8	3.4–595. 3	89.9	117.7 ± 95.8	8.3–447.8	CCL2	19.0	19.8 ± 7.4	3.9–47.0	17.5	18.6 ± 6.9	8.7–65.2
CCL26	10.5	9.1 ± 5.2	0.1–20.9	8.9	8.3 ± 4.6	0.4–18.2	CCL8	15.4	16.6 ± 8.2	3.9–72.4	15.7	15.9 ± 6.1	2.8–37.9
CX3CL1	102.2	120.2 ± 83.2	56.7–695.2	99.6	108.1 ± 47.1	50.9–519.3	CCL7	41.3	43.8 ± 11.6	29.2–103.5	39.2	41.1 ± 7.9	29.2–74.0
CXCL6	20.0	21.3 ± 8.4	10.1–47.9	21.0	22.5 ± 9.3	8.5–59.3	CCL13	17.7	23.4 ± 15.5	5.0–103.6	18.3	21.6 ± 11.9	6.9–66.0
GMCSF	5.7	5.9 ± 3.7	1.0–14.5	4.3	4.9 ± 3.6	1.0–10.6	TNF-α	14.2	15.1 ± 5.4	9.2–50.6	13.4	13.9 ± 2.8	6.4–23.6
CXCL1	149.6	155.4 ± 33.0	92.5–322.7	145.2	148.5 ± 30.6	92.5–276.6	CCL17	19.4	28.3 ± 26.5	6.4–168.6	22.5	27.9 ± 19.9	3.8–115.2
CXCL2	56.3	86.0 ± 74.1	23.5–367.9	65.4	102.1 ± 90.8	16.9–426.9	CCL25	259.0	256.2 ± 87.0	112.9–648.2	241.5	240.6 ± 69.7	112.9–545.1
CCL1	44.2	44.7 ± 12.0	26.8–83.0	42.7	42.6 ± 10.4	24.6–71.0	CXCL9	119.7	185.3 ± 422.9	50.6–4189.5	121.3	170.5 ± 359.2	68.9–3925.5
IFN-γ	1.8	1.9 ± 0.6	1.1–5.9	1.8	1.8 ± 0.4	1.1–3.6	CCL3	5.2	5.6 ± 2.6	3.8–29.2	4.9	5.1 ± 0.8	3.6–7.6
IL-1β	10.3	13.4 ± 17.5	2.5–129.0	11.6	12.9 ± 13.2	1.5–143.4	CCL15	4540.9	5893.6 ± 4562.9	1259.4–26163.1	4113.2	5262.9 ± 3497.7	893.2–21597.0
IL-2	3.5	4.1 ± 5.0	1.3–50.1	3.5	3.4 ± 1.0	1.3–5.8	CCL20	7.6	16.4 ± 56.0	3.6–512.1	7.4	8.9 ± 5.6	4.0–54.1
IL-4	9.0	9.4 ± 3.2	2.7–19.4	10.0	9.7 ± 3.3	0.9–16.5	CCL19	51.7	59.3 ± 37.3	24.9–284.8	48.3	54.6 ± 24.1	20.0–167.3
IL-6	8.1	10.1 ± 8.4	3.4–76.8	8.1	8.2 ± 2.4	3.9–15.5	CCL23	316.9	324.8 ± 155.1	19.2–681.2	339.0	332.3 ± 150.4	9.8–827.4
IL-8	8.9	10.3 ± 7.6	4.1–76.8	8.9	9.0 ± 2.5	3.9–19.3	CXCL16	372.4	384.8 ± 122.3	111.6–715.4	361.4	380.2 ± 130.7	130.1–799.8
IL-10	18.9	20.7 ± 10.4	10.1–82.0	19.9	20.4 ± 8.6	7.0–73.3	CXCL12	1164.7	1130.0 ± 305.8	423.8–2075.7	1189.1	1174.4 ± 308.5	552.3–2882.1

### Statistics

For univariate statistical analysis two sided Wilcoxon rank-sum test (Mann-Whitney U test) was used to assess statistical significance of the difference in plasma concentrations of 40 different cytokines and chemokines between endometriosis patients (i.e. also according to the different types of endometriosis) and control group of women. Results of the univariate analysis were then also corrected according to Bonferroni’s correction for multiple testing. To assess the normality of the distributions Shapiro-Wilk test was used. Fisher’s exact and Chi-square tests were used for comparison of categorical variables. Results of the descriptive analysis (i.e. patient’s clinical data) were presented as mean ± standard deviation (SD) while the concentrations of the measured proteins were presented as median and also as mean ± SD (Tables 1 and 2, respectively). Before further analysis we excluded proteins with reported out of range concentrations (i.e. GM-CSF, CXCL5). Apart from the remaining single proteins additional variables were constructed which represented ratios of the protein’s concentrations. Batch effect between samples collected in different centres was identified with principle component analysis (PCA) and removed using mean-centring and normalisation of standard deviations of all protein features across samples from each batch.

#### Machine learning

Machine learning algorithms such as decision tree^22^, generalised linear model^23^, weighted k-nearest neighbour^24^ and random forest^25^ were applied to identify proteins or panels of proteins that would discriminate patients with endometriosis from the controls. R packages *rpart*, *GLMNET*, *KKNN* and *RandomForest* were used to implement aforementioned models. Selected machine learning methods represent very popular, however, intrinsically different classes of classification algorithms. Each employed method is sufficiently simple to produce interpretable results, but at the same time powerful enough to model complex and often non-linear interactions between input features. In order to ensure robustness of the reported results, 4-fold repeated cross-validation (4-fold repeated CV) technique has been used. For each classifier average accuracy across all the folds and repetitions were reported. Reported accuracy has been compared to the accuracy of the hypothetical random classifier trained on the same data to assess the diagnostic potential of the trained models. At times when number of samples was not equal in modelled groups, balanced accuracy which takes into account imbalanced representation of samples was applied instead of regular accuracy. We have included additional clinical data into our analysis such as the use of hormonal therapy and/or oral contraception three months prior to surgery, medication intake a week prior to surgery as potential important confounders or effect modifiers. The obtained metadata are included in the Table 1 and in the Supplementary Table S1.

## Results

### Characteristics of the patient’s cohorts

Our case group comprised 116 patients with different types of endometriosis (Tables 1 and S1). Staging of endometriosis was done according to the revised American Society for Reproductive Medicine classification^3^. Minimal to mild endometriosis was present in 72 patients (62%) and moderate to severe in 40 patients (35%) and for four (3%) patients the information regarding the extent of endometriosis was not known. Patients with endometriosis were 32 ± 6 years of age (range between 19 and 50 years) and with a body mass index (BMI) of 23 ± 5 kg/m^2^ (range between 16 and 50 kg/m^2^). According to the menstrual phase 59 patients (51%) were in their secretory and 49 (42%) in their proliferative phase (Table 1), six (5%) patients were on oral contraceptives at the time of the hospitalization, and for two (2%) patients this information was missing.

Patients with benign gynaecological conditions (i.e. different types of cysts and/or myoma), unexplained infertility and/or severe pain where laparoscopy excluded the presence of endometriosis totalled 94 controls. Controls were 32 ± 8 years of age (range between 18 and 50 years) and with a BMI of 24 ± 4 kg/m^2^ (range between 18 and 42 kg/m^2^). In total 41 (44%) controls were in secretory and the same number of controls were in proliferative phase of their menstrual cycle (Table 1) while four (4%) controls were taking oral contraceptives at the time of the surgery and for eight (8%) controls the information was missing or the phase of the menstrual cycle could not be determined.

Three months prior to surgery the vast majority of our study participants was not on hormonal therapy, only 8.5% controls and 10.3% of endometriosis patients used hormonal therapy (mainly progesterone and progestins), and additional 11.7% controls and 12.9% patients with endometriosis was on oral contraception (Table 1). A week before surgery 54 patients with endometriosis (47%) and 39 controls (42%) were taking medications, mostly analgesics, anti-inflammatory and anti-rheumatic products and psychoanaleptics. More than half of the patients with endometriosis (59%) and less than a half of controls (48%) were non-smokers (Table 1). Sport or recreation two days before surgery was reported for 39 patients with endometriosis (34%) and 19 (20%) controls.

The two study groups did not differ in age, menstrual phase, use of hormonal therapy and oral contraceptives three months prior to surgery, use of other medications a week before surgery, and smoking status. However, they differed in BMI distribution (P < 0.05), frequency of dysmenorrhea (P < 0.01), intensity of dysmenorrhea (P < 0.05) and in presence of additional pathologies/conditions such as fallopian tube related pathologies (P < 0.01), cysts (P < 0.01) and inflammation related conditions (P < 0.05). Most of the study participants (59%) were of Slovene or Austrian origin and all of the participants were of European descent. This clinical information is summarized in Tables 1 and S1.

### Levels of cytokines in patients with endometriosis and in control

**Figure 2:**
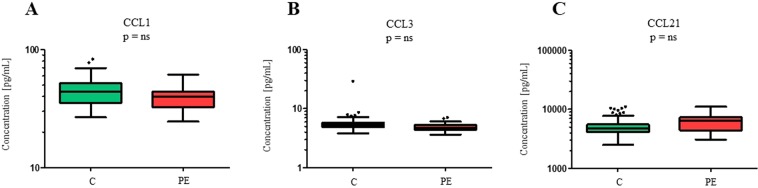
Box plots comparing plasma levels of the three cytokines that differ between the control group of patients and patients with peritoneal endometriosis in the univariate analysis. Plasma levels of cytokines are presented as Tukey box-and-whiskers plots with median, the box from the 25^th^ to 75^th^ percentiles, and whiskers correspond to the 25^th^ percentile minus 1.5 times IQR (interquartile range) and to the 75^th^ percentile plus 1.5 IQR. After correction for multiple testing no statistical difference (ns) was observed. Plasma concentrations of the cytokines are represented on a logarithmic scale. C, controls; PE, peritoneal endometriosis.

In all 210 plasma samples concentrations of all 40 different chemokines were measured (Table 2). Univariate statistical analysis revealed that there are no statistically significant differences in cytokine levels between all patients and controls. We also compared plasma concentrations of cytokines from patients with different types of endometriosis with controls where we identified eleven potential biomarkers for a specific type of endometriosis. In total we have identified seven potential biomarkers for peritoneal endometriosis (i.e. CCL21, CCL11, CCL26, CX3CL1, CCL1, IL-6, and CCL3), two for the presence of peritoneal and ovarian endometriosis (i.e. CXCL11, CXCL12), one for peritoneal and deep infiltrating endometriosis (i.e. IFN-γ) and two for all three types of endometriosis (i.e. CCL15, CXCL12). The most differential proteins for peritoneal endometriosis CCL1, CCL3 and CCL21 (Fig. 2) but after correction for multiple testing a boundary of the statistical significance was set at P < 0.001 and the differences in the concentrations of these proteins were not statistically significant.

### Analysis of cytokines by different machine learning approaches did not allow separation between cases and controls

**Figure 3:**
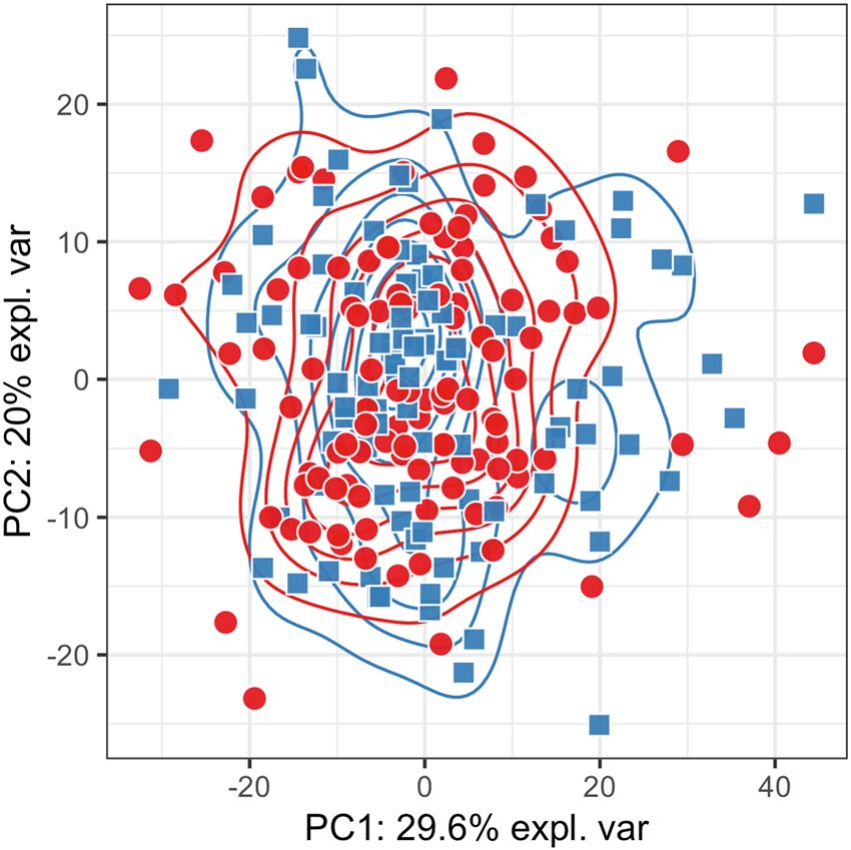
Principal component analysis plot. Data from the protein concentrations were scaled and normalized. The PCA plot is based on the whole protein set and coloured according to the disease status (red circles - patients with endometriosis; blue squares - controls). Transformed data show no meaningful grouping between patients with endometriosis and controls.

**Figure 4:**
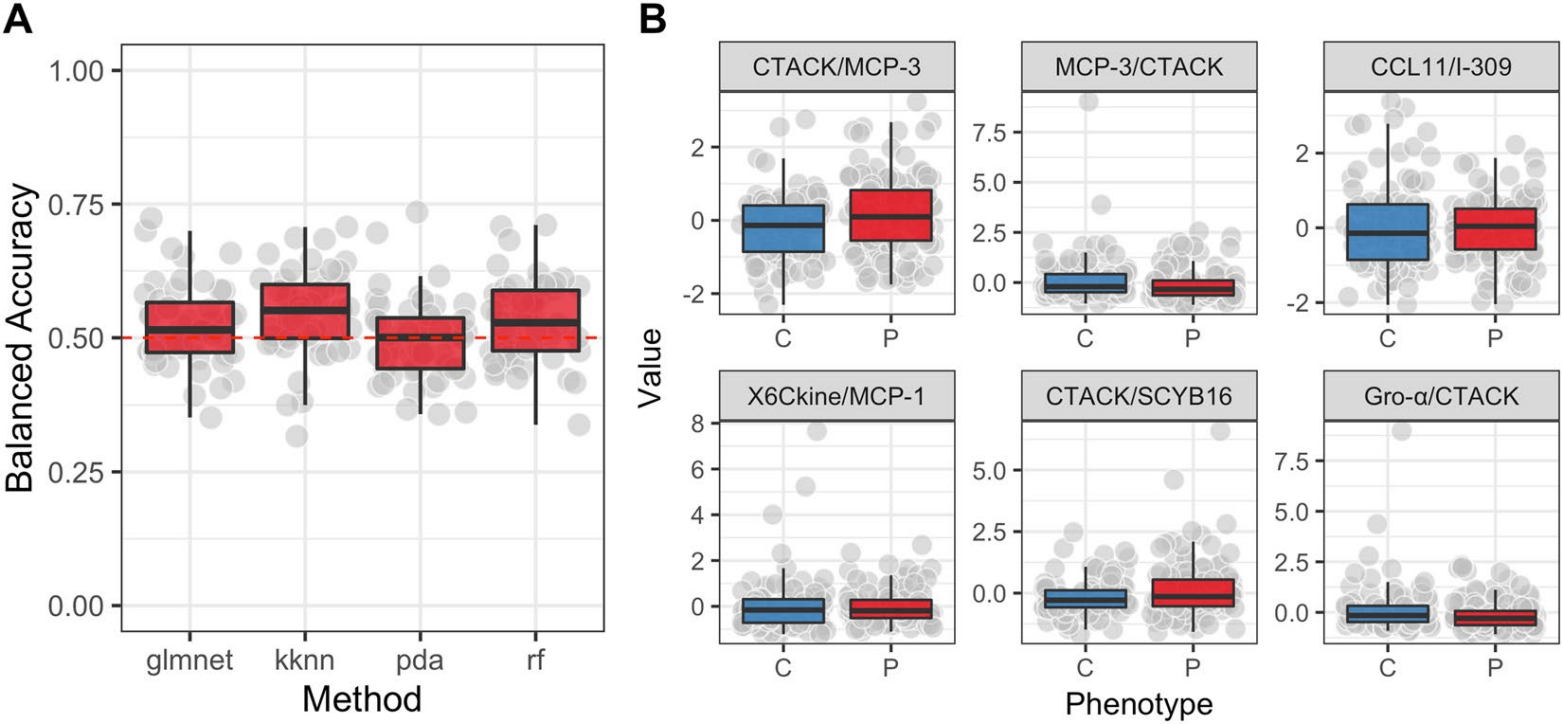
Averaged classification performance of four classifiers and box plots for the selected features that were used for training a random forest model. (**A**) Four different classifiers were used based on the data from the training set with the highest average classification performance (i.e. accuracy) achieved with random forest (balanced accuracy of ~59%). (**B**) Box plots of the six most important features that were used for training a RandomForest model based on the training set and were the most differential between patients with endometriosis and controls. Red color designate patients with endometriosis and blue color controls. Machine Learning models used: glmnet, elastic-net regularized generalized models; kknn, Weighted k-Nearest Neighbors; rpart, Recursive Partitioning and Regression Trees; rf, RandomForest. Dashed red line indicates expected balanced accuracy of a random chance.

**Figure 5:**
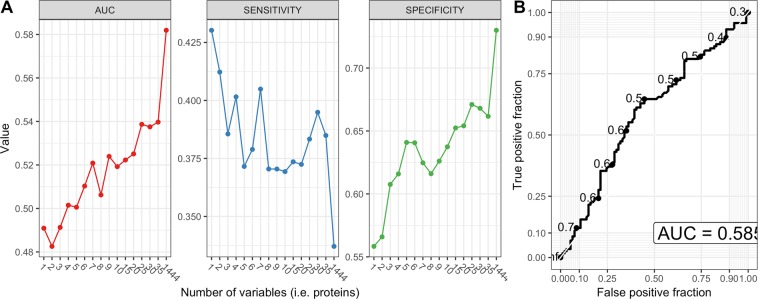
Modelling results from the recursive feature elimination method. **(A)** Each dot that forms curves was chosen automatically by the random forest algorithm trained on the number of protein features specified by x-axis. The best performance was achieved by random forest that was trained on all 1444 protein concentrations or ratios of protein concentrations remaining after pre-processing which achieved AUC of 0.585 for all samples with a sensitivity of 34% and specificity of over 70%. (**B)** ROC curve based on the highest values of sensitivity, specificity and AUC. ROC, receiver operating characteristic; AUC, area under the curve.

As more biomarkers potentially increase the reliability of a diagnostic test we decided to use machine learning to evaluate whether a panel of proteins with or without incorporation of metadata can differentiate among our two phenotypes. Results of the PCA showed there was no meaningful separation between patients with endometriosis and the controls based on the measured plasma levels of cytokines (Fig. 3). The highest average classification performance was achieved by the random forest algorithm (balanced accuracy = 59%, see Fig. 4a) with signal of six most influential features (i.e. proteins) illustrated as boxplots in Fig. 4. Obtained accuracy was not sufficiently different from random chance. Next, we trained the random forest model on different numbers of protein features to test a hypothesis that model trained on fewer proteins would generate better diagnostic characteristics (i.e. higher sensitivity, specificity and AUC) rather than using the whole panel of proteins and protein ratios at once. Results showed that a combination of three proteins would generate the highest combination of the selected diagnostic characteristics with a sensitivity of 40%, specificity of 65% and an AUC of 0.61 (Fig. 5), which, however is still far from being acceptable for diagnostics.

**Figure 6:**
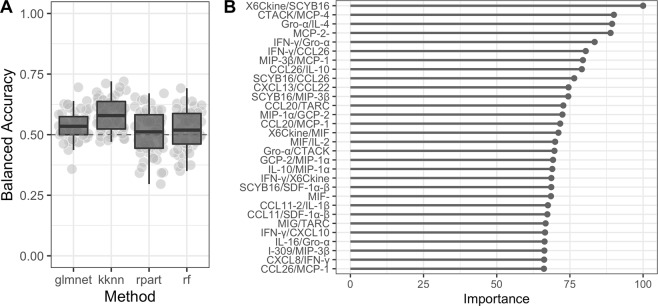
Modelling results after inclusion of metadata. **(A)** Random forest achieved the highest balanced accuracy on average (~0.55). (**B)** Both protein and metadata features ranked by their relative importance for RandomForest predictive performance. The last step was to evaluate if there is any clear separation between patients within individual types of endometriosis and controls with the inclusion of metadata. Results also showed that there is no improvement of discriminating performance of the classifiers if we look into individual type of endometriosis.

**Figure 7:**
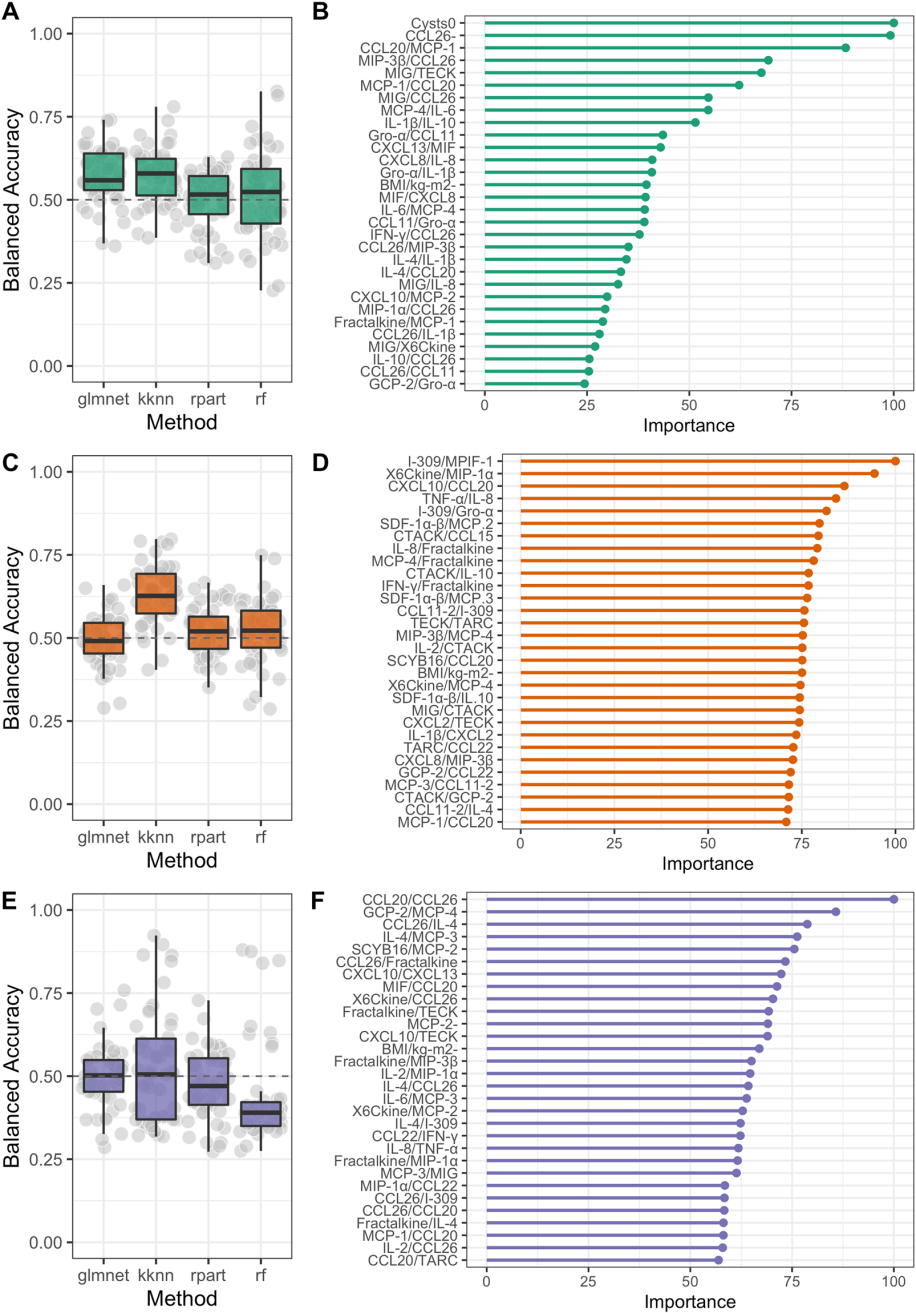
Modelling results after inclusion of metadata for individual types of endometriosis and controls. Nested Cross-validation results for three machine learning methods on patients with ovarian (**A**,**B**) peritoneal (**C**,**D**) and deep infiltrating endometriosis (**E**,**F**) and control samples. 5-fold CV was repeated 10 times without any parameter learning or sharing allowed between the folds to ensure generalisation and robustness of the obtained models. Results suggest that machine learning models cannot differentiate between different types of endometriosis and controls with an accuracy that exceeds the one of random chance.

We then added metadata features (included in Tables 1 and S1) along with protein levels to the training data, but it did not improve the overall fit of the models and we could still not see a clear separation between patients with endometriosis and controls (Fig. 6). Performing separate analysis on individual types of endometriosis with the inclusion of metadata revealed no significant differences (Fig. 7). Comparing all four stages (minimal, mild, moderate and severe) of endometriosis as well as comparing minimal/mild with moderate/severe endometriosis with controls did not end up in significantly different features. Except for TNFα/CCL27 protein ratio that has been consistently reported by the random forest algorithm as the most valuable feature for separating patients with minimal/mild endometriosis from controls. However, despite high importance of TNFα/CCL27, the accuracy achieved by the algorithm remained modest (57.4%). We also did not observe any significant differences between patients and controls when divided with respect to the medication intake (use of any kind of medication or the use of nonsteroidal anti-inflammatory drugs or the use of any type of hormonal medication). Other personal and clinical data also showed no differences in the plasma profiles of the patients and controls.

## Discussion

Endometriosis is a common benign gynaecological condition that is characterized by the presence of endometrial lesions in the peritoneal cavity and is thus also described as a chronic inflammatory disease where diagnostic biomarkers that would be applicable for clinical use have not yet been identified^16^. Cytokines have already been investigated as potential biomarkers of endometriosis in the blood and/or peritoneal fluid. In addition to individual inflammatory proteins, also panels of cytokines in conjunction with other proteins have been studied, although the results of these studies varied^16,19,26^. The majority of these studies investigated IL-6 and INF-γ which are included in the activation and differentiation of inflammatory cells and are also involved in the pathogenesis of endometriosis^27–30^.

In the current study there were no statistically significant differences in cytokine plasma levels between patient with different types of endometriosis and controls. Although we found potential importance of the ratio TNFα/CCL27 for separating patients with minimal/mild endometriosis from the control group of patients, the accuracy achieved by the algorithm was insufficient. Univariate analysis revealed the lowest P values when we compared concentrations of cytokines/chemokines CCL1, CCL3, CCL21 in patients with peritoneal endometriosis and controls. We found no published studies evaluating blood levels of these cytokines/chemokines in patients with endometriosis Borrelli *et al*., evaluated the levels of CCL21 in the peritoneal fluid of 36 patients with endometriosis and 27 controls and reported no significant differences^31^. Other studies focused on the expression of the corresponding genes. Unchanged or changed expression (i.e. increased/decreased) in the endometrium from patients with endometriosis was reported for *CCL21* with no explanation on how these changes might contribute to the aetiology or pathogenesis of endometriosis^32–34^. Expression and/or role of CCL1 and its receptor (i.e. CCR8) in endometrial tissue was studied by Shi *et al*.^35,36^ and revealed higher expression and their potential role in the pathogenesis of endometriosis. Although these three cytokines/chemokines identified in our study have so far not been sufficiently investigated in endometriosis our experimental data link changes in concentrations of these proteins to peritoneal endometriosis, which implies that CCL1, CCL3 and CCL21 might have a role in the aetiology and pathogenesis of this type of endometriosis.

The studies that evaluated blood concentrations of cytokines and chemokines as potential biomarkers of endometriosis are scarce, as the most studies so far evaluated the diagnostic potential of inflammatory proteins in peritoneal fluid and/or tissue samples (i.e. eutopic/ectopic endometrium) of patients with endometriosis. Kalu *et al*., evaluated a panel of 10 cytokines in peritoneal fluid and serum of women undergoing laparoscopy for unexplained infertility. Their study group comprised of women with minimal or mild endometriosis that were compared to the control group of women with unexplained infertility and absence of endometriosis. Elevated levels of CCL2, IL-8 and IL-6 were found in peritoneal fluid while the equivalent increase in serum samples was not found^37^. Similar study was later on conducted by Hassa *et al*. that evaluated the diagnostic potential of four cytokines (IL-2, IL-4, IL-10, IFN-γ) and immune cells in serum and peritoneal fluid of patients with endometriosis comparing to healthy group of patients^38^. No significant differences were observed when comparing control group with early and late stageendometriosis patients. Recently, Fan *et al*. evaluated seven cytokines including IL-10, IL-6, IL-4 and IL-2 in serum and peritoneal fluid from endometriosis patients and control patients and found significantly higher levels of IL-10 and lower levels of IL-2 in serum, but significantly higher levels of IL-2 in peritoneal fluid^39^. Amongstudies evaluating cytokines as blood biomarkers Rocha *et al*.^40^ followed a criteria for case-control studies where case and control groups originate from the same cohort^41^. In their study all of the patients presented with at least one endometriosis-like symptom (i.e. chronic pelvic pain and/or infertility and/or potential presence of endometrioma based on the ultrasound). After the laparoscopic operation and histological evaluation patients were divided into two groups; patients with endometriosis (n = 44) and control group of patients (n = 31). Concentrations of seven different cytokines (i.e. IL-2, IL-4, IL-6, IL-10, CCL2, CXCL10, and CCL11) were simultaneously determined using cytometric bead array and results showed that based on the panel of these cytokines and clinical data it was not possible to predict the presence of endometriosis in a group of symptomatic patients^40^.

Recently, Aalamat *et al*. published a systematic review on the use of multiplex technology for identification of potential novel biomarkers of endometriosis among inflammation associated proteins^42^. They reported that the majority of studies that adapted multiplex technology evaluated potential novel biomarkers of endometriosis in peritoneal fluid^20,31,43–47^. Although peritoneal fluid is collected by a semi-invasive method it is the most representative sample that closely reflects inflammatory changes that are associated with the pathogenesis of endometriosis^48^. Based on the literature and our published studies^20^, we conclude that cytokines and chemokines in peritoneal fluid have a far greater diagnostic potential for endometriosis than their plasma or serum concentrations. Results of our study are also in concordance with the study conducted by Lee *et al*. that evaluated the diagnostic potential of pro-inflammatory oxylipins and cytokines in serum samples of 103 women undergoing laparoscopy. Results of their study showed limited diagnostic potential of the measured circulating biomarkers for the diagnosis of endometriosis, warranting additional studies to evaluate the exact role of systemic inflammation in endometriosis^49^.

Although we evaluated a broad spectrum of inflammatory proteins in plasma samples of patients with different types of endometriosis and controls with several different multifactorial benign gynaecological conditions, both within a well-defined cohort, included detailed protocols, obtained a large set of clinical data, included different nationalities, combined with high throughput methodology and advanced statistical approaches, our results were consistent with several previous studies indicating limited diagnostic potential of circulating cytokines for the diagnosis of endometriosis. Having said this, presented results need to be considered carefully as they might be subject to various sources of bias and noise. Self-reporting of metadata by patients, undetected technical batch effects, unpredictable statistical fluctuations are all potential sources of bias and thus, limiting factors of the current study.

## Conclusions

In this study we evaluated the diagnostic potential of 40 different cytokines in plasma samples from 210 patients with different types of endometriosis and control group of patients from two medical centres. Although several studies have associated inflammation with the development and progression of endometriosis, and inflammatory cytokines in endometrial tissue, peritoneal fluid and blood have been evaluated as potential biomarkers for endometriosis, the published results are inconsistent and identified no clinically useful biomarker to date. Based on the evaluated plasma concentrations of these 40 different cytokines, clinical data and appropriate statistical analysis we were unable to develop a diagnostic algorithm that would separate patients with endometriosis from the control group of patients with sufficient sensitivity and specificity. For development of a model with potential clinical applicability, which would enable diagnosis of patients with endometriosis with sufficient accuracy, further approaches of targeted and non-targeted “omics” technologies will be needed in conjunction with appropriate statistical/bioinformatics methods. These have to be followed by independent validation studies to confirm the results obtained in a research setting.

## Supplementary information

Table S1: Detailed clinical characteristics of the study participants.

## Data Availability

All data are fully available without restriction.
