# Supplementary material for: Multiplex analysis of 40 cytokines do not allow separation between endometriosis patients and controls
Source: Sci Rep. 2019 Nov 13;9:16738. doi: 10.1038/s41598-019-52899-8 (PMC6853932; doi:10.1038/s41598-019-52899-8)
Supplement: Supplementary file 1 — Table S1: Detailed clinical characteristics of the study participants. [file 41598_2019_52899_MOESM1_ESM.docx]

**Supplementary Information**

**Multiplex analysis of 40 cytokines do not allow separation between endometriosis patients and controls**

Tamara Knific^#1^, Dmytro Fishman^#2,3^, Andrej Vogler^4^, Manuela Gstöttner^5^, René Wenzl^5^, Hedi Peterson^2,3^, and Tea Lanišnik Rižner^1^

*^1^Institute of Biochemistry, Faculty of Medicine, University of Ljubljana, 1000 Ljubljana, Slovenia*

*^2^Institute of Computer Science, University of Tartu, Liivi 2, 50409 Tartu, Estonia*

*^3^Quretec Ltd., Ülikooli 6A, Tartu 51003, Estonia*

*^4^Department of Obstetrics and Gynaecology, University Medical Centre Ljubljana, 1000 Ljubljana, Slovenia*

*^5^Department of Obstetrics and Gynecology, Medical University Vienna, 1090 Vienna, Austria*

# Contributed equally

**Table S1:** **Detailed clinical characteristics of the study participants.**

|  | Centre | Slovenia | | | | Austria | | | |
| --- | --- | --- | --- | --- | --- | --- | --- | --- | --- |
| Characteristic | Subgroup | Controls n = 47 | | Patients n = 78 | | Controls n = 47 | | Patients n = 38 | |
|  |  | Frequency | [%] | Frequency | [%] | Frequency | [%] | Frequency | [%] |
| **Age (years)** | <26 | 8 | 17.0 | 8 | 10.3 | 8 | 17.0 | 8 | 21.1 |
|  | 26-29.9 | 15 | 31.9 | 16 | 20.5 | 5 | 10.6 | 7 | 18.4 |
|  | 30-35.9 | 17 | 36.2 | 41 | 52.6 | 9 | 19.2 | 7 | 18.4 |
|  | 36-40.9 | 7 | 14.9 | 13 | 16.7 | 14 | 29.8 | 10 | 26.3 |
|  | >41 | 0 | 0 | 0 | 0 | 11 | 23.4 | 6 | 15.8 |
| **BMI (kg/m^2^)** | Underweight < 18.5 | 1 | 2.1 | 5 | 6.4 | 2 | 4.3 | 6 | 15.8 |
|  | Normal 18.6-24.9 | 32 | 70.2 | 56 | 71.8 | 28 | 59.6 | 23 | 60.5 |
|  | Overweight 25-29.9 | 11 | 21.3 | 12 | 15.4 | 13 | 27.7 | 3 | 7.9 |
|  | Obese > 30 | 3 | 6.4 | 5 | 6.4 | 4 | 8.5 | 6 | 15.8 |
| **Menstrual phase** | Proliferative | 24 | 51.1 | 36 | 46.2 | 17 | 36.2 | 13 | 34.2 |
|  | Secretory | 21 | 44.7 | 38 | 48.7 | 20 | 42.6 | 20 | 52.6 |
|  | Oral contraceptives | 2 | 4.3 | 3 | 3.9 | 2 | 4.3 | 3 | 7.9 |
|  | Irregular/anovulatory | 0 | 0 | 0 | 0 | 2 | 4.3 | 0 | 0 |
|  | Missing | 0 | 0 | 1 | 1.3 | 6 | 12.8 | 2 | 5.3 |
| **Ethnicity** | Native | 32 | 68.1 | 57 | 73.1 | 19 | 40.4 | 16 | 42.1 |
|  | Native-foreign | 4 | 8.5 | 10 | 12.8 | 5 | 10.6 | 3 | 7.9 |
|  | Foreign | 9 | 19.2 | 10 | 12.8 | 23 | 48.9 | 19 | 50.0 |
|  | Missing | 2 | 4.3 | 1 | 1.3 | 0 | 0 | 0 | 0 |
| **Smoking status** | Regular | 14 | 29.8 | 18 | 23.1 | 17 | 36.2 | 11 | 29.0 |
|  | Occasional | 2 | 4.3 | 4 | 5.1 | 3 | 6.4 | 2 | 5.3 |
|  | Former | 6 | 12.8 | 7 | 9.0 | 6 | 12.8 | 5 | 13.2 |
|  | Non-smoker | 24 | 51.1 | 48 | 61.5 | 21 | 44.7 | 20 | 52.6 |
|  | Missing | 1 | 2.1 | 1 | 1.3 | 0 | 0 | 0 | 0 |
| **Oral contraceptives 3 months before surgery** | No | 44 | 93.6 | 71 | 91.0 | 39 | 83.0 | 30 | 79.0 |
|  | Yes | 3 | 6.4 | 7 | 9.0 | 8 | 17.0 | 8 | 21.1 |
| **Hormonal therapy 3 months before surgery** | No | 42 | 89.4 | 68 | 87.2 | 44 | 93.6 | 36 | 94.7 |
|  | Yes | 5 | 10.6 | 10 | 12.8 | 3 | 6.4 | 2 | 5.3 |
| **Medications a week before surgery** | No | 35 | 74.5 | 50 | 64.1 | 20 | 42.6 | 12 | 31.6 |
|  | Yes | 12 | 25.5 | 28 | 35.9 | 27 | 57.5 | 26 | 68.4 |
| **Additional pathologies/conditions** | Fallopian tube related | 6 | 12.8 | 1 | 1.3 | 9 | 19.2 | 2 | 5.3 |
|  | Cysts | 13 | 27.7 | 8 | 10.3 | 18 | 38.3 | 6 | 15.8 |
|  | Adhesions | 13 | 27.66 | 26 | 33.3 | 1 | 2.1 | 0 | 0 |
|  | Inflammation related | 7 | 14.9 | 1 | 1.3 | 4 | 8.5 | 2 | 5.3 |
|  | Uterus related | 2 | 4.3 | 9 | 11.5 | 6 | 12.8 | 0 | 0 |
|  | Adenomyosis | 0 | 0 | 1 | 1.3 | 2 | 4.3 | 2 | 5.3 |
|  | Borderline ovarian tumor | 0 | 0 | 0 | 0 | 2 | 4.3 | 0 | 0 |
| **Type of endometriosis** | Ovarian |  |  | 6 | 7.7 |  |  | 10 | 26.3 |
|  | Peritoneal |  |  | 42 | 53.9 |  |  | 5 | 13.2 |
|  | Deep infiltrating |  |  | 3 | 3.9 |  |  | 1 | 2.6 |
|  | Ovarian, peritoneal |  |  | 12 | 15.4 |  |  | 10 | 26.3 |
|  | Ovarian, deep infiltrating |  |  | 3 | 3.9 |  |  | 3 | 7.9 |
|  | Ovarian, peritoneal, deep infiltrating |  |  | 10 | 12.8 |  |  | 6 | 15.8 |
|  | Peritoneal, deep infiltrating |  |  | 2 | 2.6 |  |  | 3 | 7.9 |
|  | Missing |  |  | 0 | 0 |  |  | 0 | 0 |
| **rAFS stage** | Minimal - mild  (I - II) |  |  | 50 | 64.1 |  |  | 22 | 57.9 |
|  | Moderate - severe (III - IV) |  |  | 28 | 35.9 |  |  | 12 | 31.6 |
|  | Missing |  |  | 0 | 0 |  |  | 4 | 10.5 |
